# Identification of a basement membrane-related genes signature to predict prognosis, immune landscape and guide therapy in gastric cancer

**DOI:** 10.1097/MD.0000000000035027

**Published:** 2023-09-29

**Authors:** Zhi-Yang Liu, Lin Xin

**Affiliations:** a Department of General Surgery, Second Affiliated Hospital of Nanchang University, Nanchang, China.

**Keywords:** basement membrane, gastric cancer, immunity, nomogram, signature, therapeutic response

## Abstract

The basement membrane is an essential defense against cancer progression and is intimately linked to the tumor immune microenvironment. However, there is limited research comprehensively discussing the potential application of basement membrane-related genes (BMRGs) in the prognosis evaluation and immunotherapy of gastric cancer (GC). The RNA-seq data and clinical information of GC patients were collected from the TCGA and GEO database. Prognosis-associated BMRGs were filtered via univariate Cox regression analysis. The 4-BMRGs signatures were constructed by lasso regression. Prognostic predictive accuracy of the 4-BMRGs signature was appraised with survival analysis, receiver operating characteristic curves, and nomogram. Gene set enrichment analysis (GSEA), gene ontology, and gene set variation analysis were performed to dig out potential mechanisms and functions. The Estimate algorithm and ssGSEA were used for assessing the tumor microenvironment and immunological characteristics. Identification of molecular subtypes by consensus clustering. Drug sensitivity analysis using the “pRRophetic” R package. Immunotherapy validation with immunotherapy cohort. A 4-BMRGs signature was constructed, which could excellently predict the GC patient prognosis (5-year AUC value of 0.873). Kaplan–Meier and Cox regression analyses showed that the 4-BMRGs signature was an OS-independent prognostic factor, and that higher risk scores were associated with shorter OS. The high-risk subgroup exhibits a higher abundance of immune cell infiltration, such as macrophages. Additionally, we observed a strong correlation between 2 BMRGs (LUM, SPARC) and immune cells such as CD8 + T cells and macrophages. The high-risk subgroup appears to be more sensitive to Axitinib, DMOG, Gemcitabine and Docetaxel by pRRophetic analysis. Furthermore, the validation of the cohort that received immune therapy revealed that patients in the high-risk group who underwent immune checkpoint inhibitor treatment exhibited better response rates. Pan-cancer analysis also shows that risk scores are strongly associated with immune and carcinogenic pathways. The 4-BMRGs signature has demonstrated accuracy and reliability in predicting the GC patient’s prognosis and could assist in the formulation of clinical strategies.

## 1. Introduction

Gastric cancer (GC) is an extremely aggressive malignant tumor around the world. The latest statistics indicate stomach cancer deaths exceed 760,000 cases.^[1]^ Despite rapid progress in the detection and treatment of GC in recent years, it still ranks 5th globally in new cases and 4th in deaths.^[1]^ Most GC patients are not diagnosed until the middle to late clinical stage as there are no specific symptoms in the early stages.^[2]^ Endoscopy is effective in detection, but it is invasive and has many adverse effects.^[3]^ Although some new technical methods, such as deep learning and sensors, have been applied to the research and application of clinical diagnosis,^[4–6]^ their accuracy and convenience still cannot fully meet the clinical needs. Consequently, identifying effective prognostic biomarkers for GC is crucial to the formulation of treatment strategies and improvement of patient outcomes.

The basement membrane is a layer of extracellular matrix (ECM) rich in collagen (IV) and laminin that serves as an important histologically identifiable barrier between carcinoma in situ and invasive cancer.^[7]^ Differences between non-fatal neoplastic lesions and invasive cancers with associated high mortality rates, whether they break through the basement membrane or not.^[7]^ Endoscopic treatment alone or surgery is potentially curative for patients with early gastric cancer while the survival rate for patients with metastatic gastric cancer is less than 30%.^[8,9]^ Research indicates that the basement membrane is disrupted in invasive gastric cancer, particularly the linearity of type IV collagen, and that MMP2 expression is elevated near the basement membrane.^[10]^ When BM is dysregulated, however, it can promote tumor migration and invasion.^[11]^ Recent studies have reported that basement membranes interact with the immune microenvironment to promote tumor progression.^[12]^

As of now, prognostic features related to the basement membrane have been established with high predictive accuracy in liver cancer,^[13]^ breast cancer,^[14]^ and lung adenocarcinoma.^[15]^ However, research in this area regarding gastric cancer (GC) is limited. Therefore, in this study, we employed bioinformatics methods to construct prognostic features associated with the basement membrane in GC patients. Additionally, we explored the correlation between basement membrane related genes (BMRGs) and immune infiltration, immunotherapy, and drug sensitivity, aiming to investigate their potential mechanisms and prognostic value in gastric cancer.

## 2. Materials and methods

### 2.1. Acquisition of patient information and gene sets

Download the transcripts per kilobase of exon model per million mapped reads data from the Cancer Genome Atlas (TCGA) database (https://portal.gdc.cancer.gov/) for 375 stomach adenocarcinomas (STAD) and 32 paraneoplastic tissues. Subsequently, we downloaded the clinical information of 433 gastric cancer patients from the TCGA database and removed patients with unknown survival information to obtain clinical data of 348 patients matched with the transcriptome data. GSE62254 (n = 300, GPL570), GSE15459 (n = 192, GPL570), and GSE34942 (n = 57, GPL570) from the GEO database (https://www.ncbi.nlm.nih.gov/geo/) were used for external validation, and GSE67501, GSE126044 were used for validation of immunotherapy. The original “CEL” files for each dataset were downloaded and normalized and de-contextualized using the “ram” method in the “affy” package for the 3 microarray dataset from the GPL570 platform.^[16]^ After merging the 3 datasets, the “ combat” function in the “sva” package was used to remove batch effects for subsequent studies.^[17]^ We downloaded the normalized data directly from the GEO database for microarray data from other platforms.

Pan-cancer cohort from TCGA database (https://portal.gdc.cancer.gov/, http://api.gdc.cancer.gov/), with matching survival data from XENA (https://xenabrowser.net/).

In addition, we collected expression data and clinical information for the immunotherapy cohort IMvigor210 cohort for subsequent analysis.

### 2.2. Identification of differentially expressed BMRGs

In the prior research, we extracted 224 BM genes. Subsequently, the “limma” R package was applied to sieve for differentially expressed BMRGs between STAD and paraneoplastic tissues with a threshold of |logFC| ≥ 1 and *P* < .05.^[18]^ Differential BMRGs were visualized using heatmap and volcano plot.

### 2.3. Construction and verification of BMRGs signature

Preliminary filtering of prognosis-related BMRGs was performed by univariate Cox in the whole cohort of GC patients. Then, the tumor patients with survival information were equally divided into 2 groups (training and validation set). The BMRGs as independent prognostic factors were screened using lasso regression analysis on the training set, and risk scores (RS) were generated for each GC patient on the basis of the corresponding regression coefficients and expressions. The formula is as follows:


$$\begin{array}{l} Risk\;score=\overset{n}{\underset{i=0}{\sum }}\left(Coef\left(i\right)\times Exp\left(i\right)\right) \end{array}$$

Stratifying patients into high- and low-risk subgroups using the median RS value of the training set as a boundary. The predictive capability of the 4-BMRGs signature on overall survival (OS) was appraised with Kaplan–Meier (KM) curves, Cox regression analysis, and receiver operating characteristic (ROC) curves.

### 2.4. Nomogram construction and validation

To construct a nomogram based on clinicopathological factors and RS utilizing the R packages “rms,” “regplot,” and “survival” to predict the GC patient’s OS. The accuracy of the nomogram was verified by graphing 5-year calibration curves. A decision curve analysis (DCA) curve is plotted to calculate the net benefit and assess the clinical applicability of the nomogram. Finally, chi-square tests were applied to assess whether clinicopathological characteristics differed between risk strata.

### 2.5. Tumor microenvironment and immune landscape analysis

The immune cell infiltration, tumor purity, and stromal cell ratio were evaluated for each GC patient utilizing the “ESTIMATE” algorithm. Single sample gene set enrichment analysis (ssGSEA) was performed on each patient to assess the degree of different immune cell infiltration using the R packages “GSEABase” and “GSVA.” The correlations between the prognostic signatures of the 4 BMRGs and immune cells were evaluated by spearman correlation analysis, which was visualized in a heatmap. Infiltration estimation profiles of all TCGA tumors downloaded from the TIMER 2.0 dataset were used to estimate the immune infiltration status of patients using different software including XCELL, CIBERSORT, MCPCOUNTER, EPIC, TIMER, and CIBERSORT-ABS.

### 2.6. Immune checkpoint expression and correlation analysis

The expression of immune checkpoint genes in different subgroups was compared using the Wilcoxon test, and violin plot was applied to illustrate the differentially expressed immune checkpoint genes.

### 2.7. Functional and pathway enrichment analysis

32 prognosis-related BMRGs were analyzed for gene ontology (GO) terms and KEGG with the “ClusterProfiler” R package. Based on the gene set “h.all.v7.5.1.symbols.gmt” retrieved from the MSigDB, gene set enrichment analysis (GSEA) was performed among different risk subsets using GSEA software (version 4.2.2). The threshold values were *P* value < .05 and FDR q value < 0.25. Gene set variation analysis (GSVA) was based on the “c2.cp.kegg.v7.5.1.symbols.gmt” gene set and visualized with a heatmap.

### 2.8. Consensus clustering based on 4-BMRGs

The “ConsensuClusterPlus” package was used to perform consensus clustering to classify STAD clusters into different clusters based on 4-BMRGs and to compare survival, immune infiltration, and immune checkpoint differences between the different subtypes.

### 2.9. Chemotherapy and immunotherapy

In addition, drug resistance is one of the barriers to oncology treatment. To assess the relationship between RS and antitumor drug sensitivity, the half maximal inhibitory concentration (IC50) of 9 drugs for each GC patient was calculated with the “pRRophetic” R package.

### 2.10. Statistical analysis

Two-sided *P* < .05 was considered statistically significant (*P < .05, **P < .01, ***P < .001). Visualization and statistical analysis were done in R 4.1.3 software.

## 3. Results

### 3.1. BMRGs filtering

**Figure 1.**
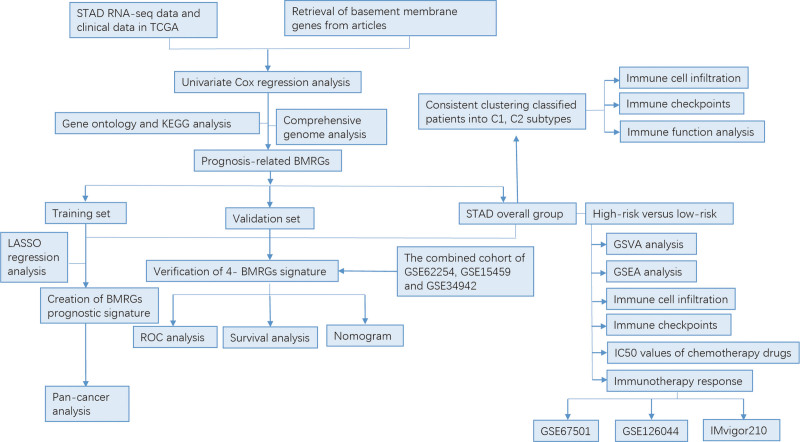
Research flow.

**Figure 2.**
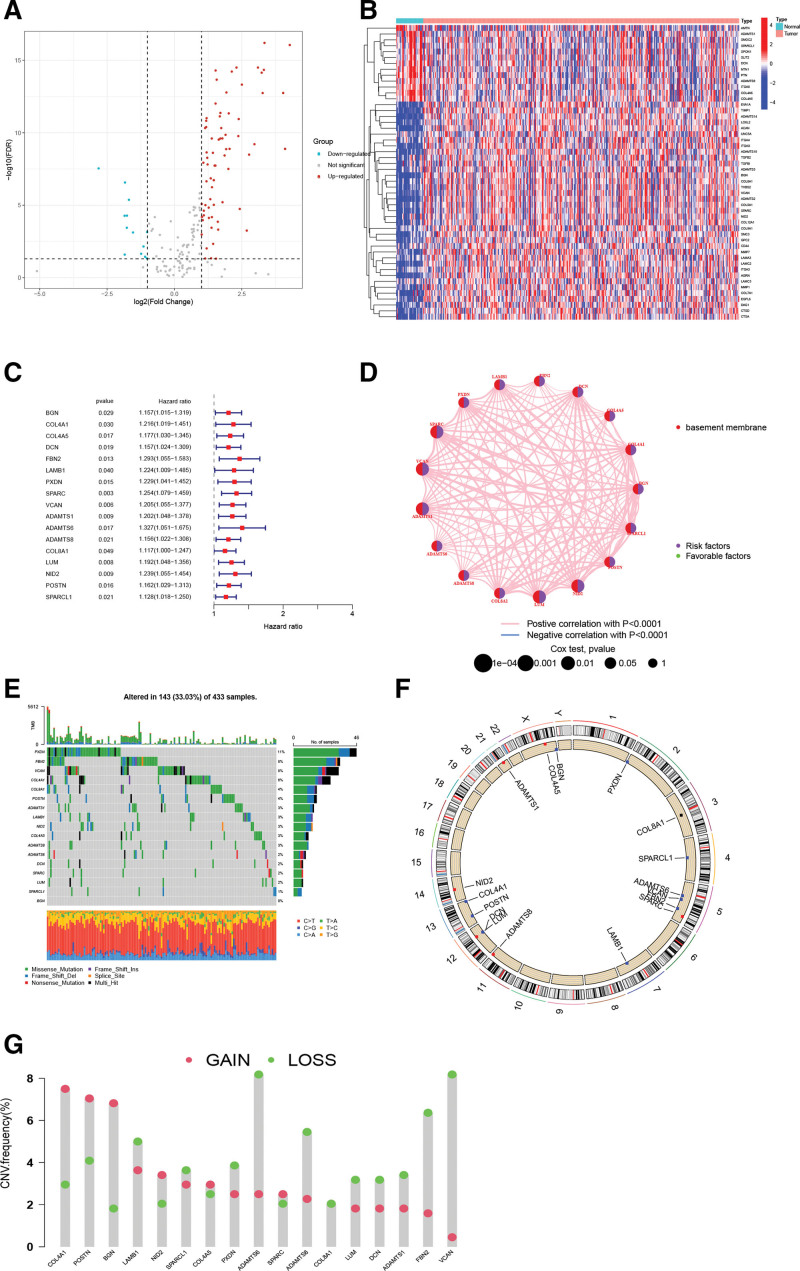
Comprehensive analysis of basement membrane-related genes. (A) Volcano map. (B) Heatmap showing the top 50 high and low expression BMRGs. (C) Univariate cox analysis. (D) Prognostic network relationship map. (E) Somatic mutations. (F) Mutation circle map. (G) CNV analysis.

The process of the study is shown in Figure 1. We firstly retrieved transcriptome data of STAD from the TCGA database. Then 224 BMRGs were obtained from the prior research (Table S1, Supplemental Digital Content, http://links.lww.com/MD/J838). 84 BMRGs were sieved by differential expression analysis based on |log2(FC)| ≥ 1, and a heatmap and volcano map are shown in Figure 2A and B.

### 3.2. Prognosis-related basement membrane genes mutation landscape and functional enrichment analysis

**Figure 3.**
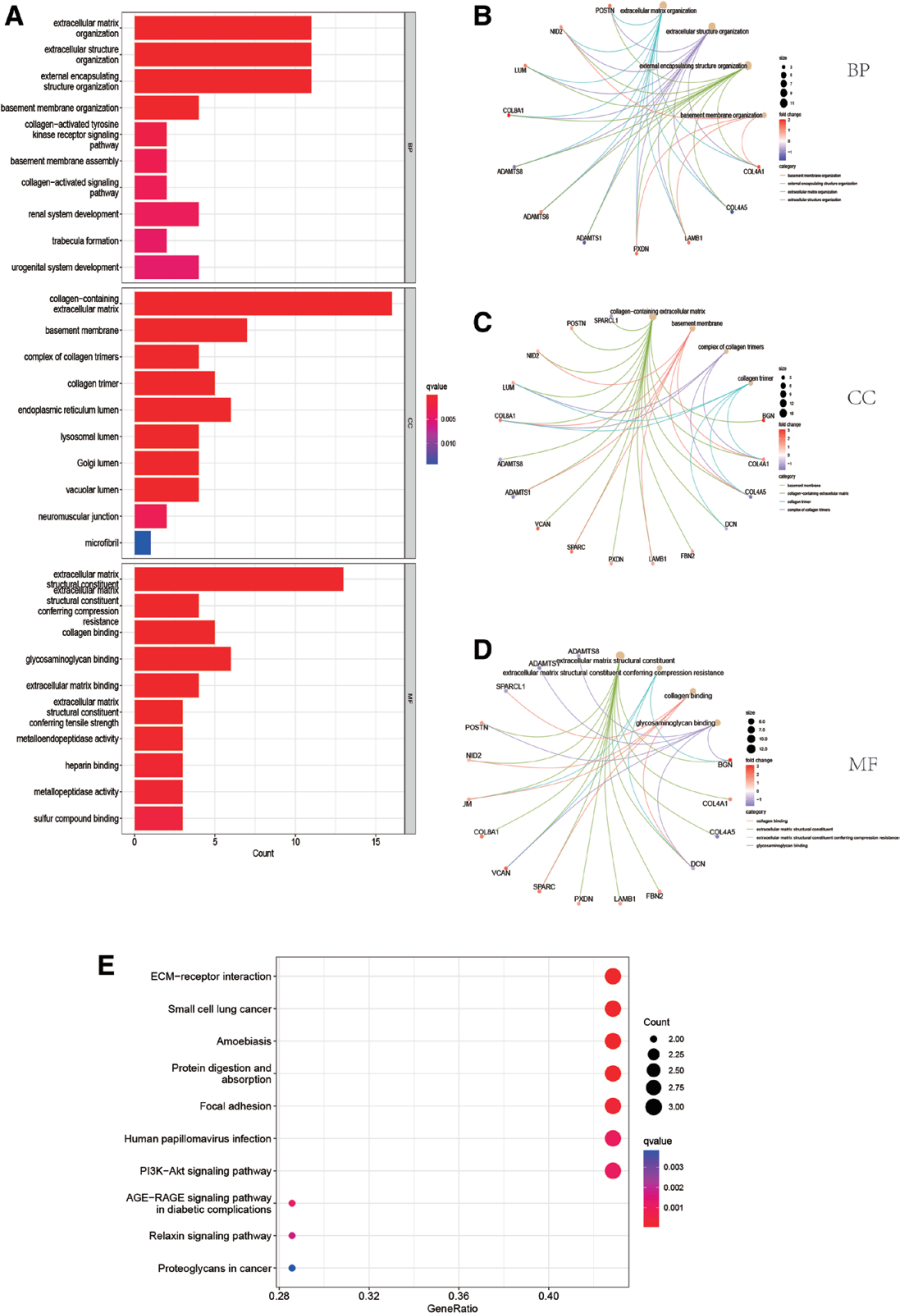
GO and KEGG analysis. (A–D) Go analysis of basement membrane prognosis-related genes. (E) KEGG analysis of basement membrane prognosis-associated genes.

Through univariate COX analysis of differentially expressed basement membrane-related genes, we obtained 17 prognosis-associated basement membrane genes and analyzed them for follow-up (Fig. 2C). First we show the prognosis-related basement membrane genes somatic mutations by plotting waterfall plots (Fig. 2E). As shown 33.03% of STAD patients had mutations in these genes, with PXDN (11%), FBN2 (8%), and VCAN (8%) being the top 3 genes in terms of mutation frequency. CNV indicates that all of these genes have amplifications and deletions (Fig. 2F and G). The prognostic correlation network revealed a strong correlation between these genes, suggesting that there may be an interaction between these basement membrane genes (Fig. 2D). The GO analysis showed that “ extracellular matrix organization,” “collagen-containing extracellular matrix” and “extracellular matrix structural constituent” are the most commonly used biological terms for biological processes, cellular components and molecular functions (Fig. 3A–D). KEGG analysis revealed that these genes were enriched in oncogenic pathways such as “ECM-receptor interaction,” “Focal adhesion” and “PI3K-Akt signaling pathway” (Fig. 3E). All these results suggest that basement membrane-associated genes are important drivers of tumor malignancy.

### 3.3. Construction and validation of BMRGs signature

**Figure 4.**
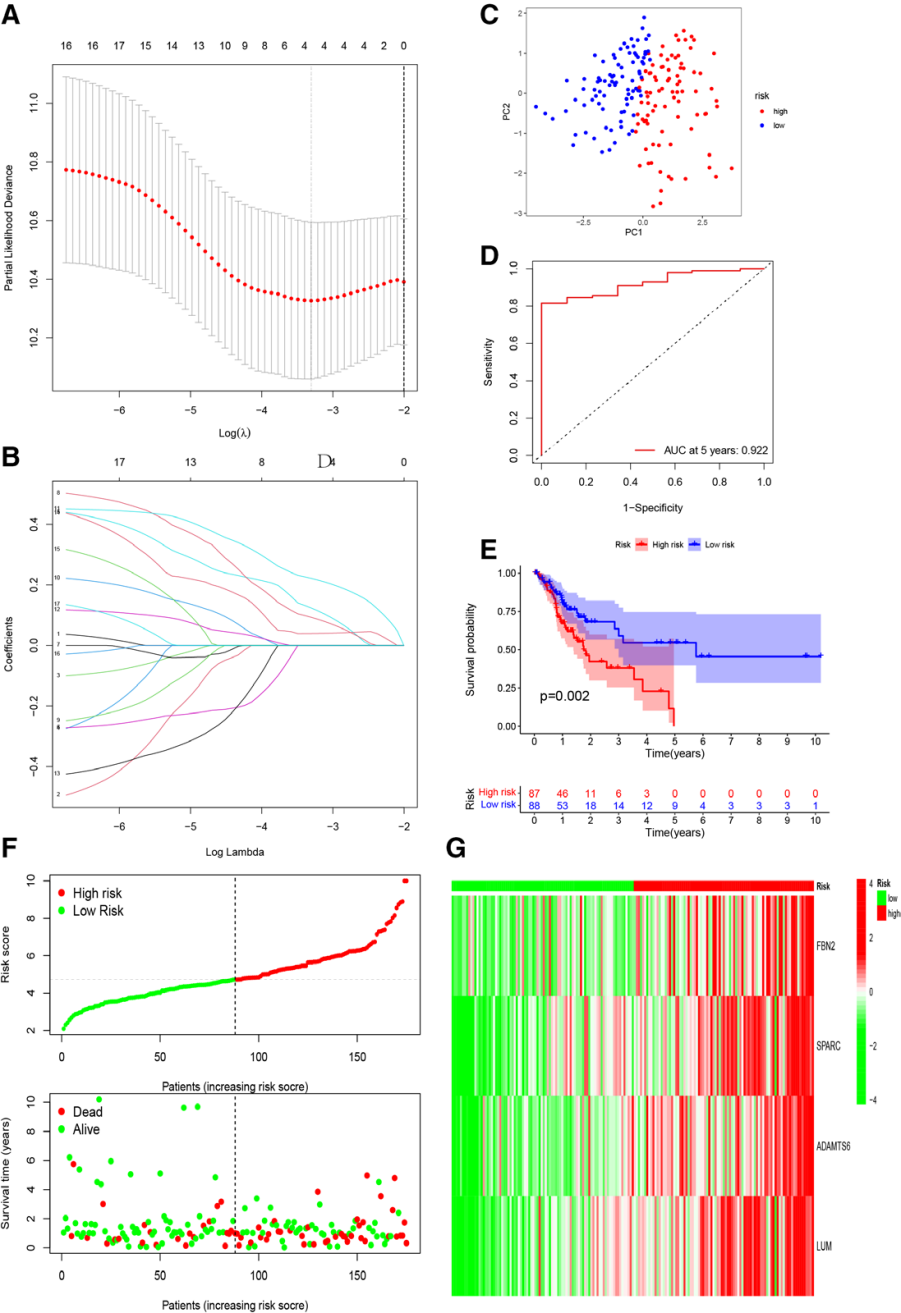
Establishment of basement membrane-associated gene prognostic signature: (A, B) training set lasso regression to determine 4-BMRGs. (C) training set PCA analysis, (D) ROC curves in the training set. (E) Kaplan–Meier survival curve for different risk subgroups in the training set. (F) survival status and risk score curves in the training set. (G) heat map of 4-BMGRs distribution in the training set. BMRGs = basement membrane related genes, ROC = receiver operating characteristic.

To construct a more accurate prediction model, 348 tumor patients with survival information were randomly and equally split into training and validation sets. The 4 BMRGS most associated with prognosis were finally identified by lasso regression analysis in the training cohort: FBN2, ADAMS6, SPARC, LUM, and the corresponding regression coefficients were obtained (Fig. 4A and B). The prognostic signature was structured based on these 4 BMRGs and the formula for calculating RS for each patient was as follows: RS = (0.123880238975713 × FBN2) + (0.0928799270728927 × SPARC) + (0.221694318455267 × ADAMTS6) + (0.0395652531540321 × LUM)

**Figure 5.**
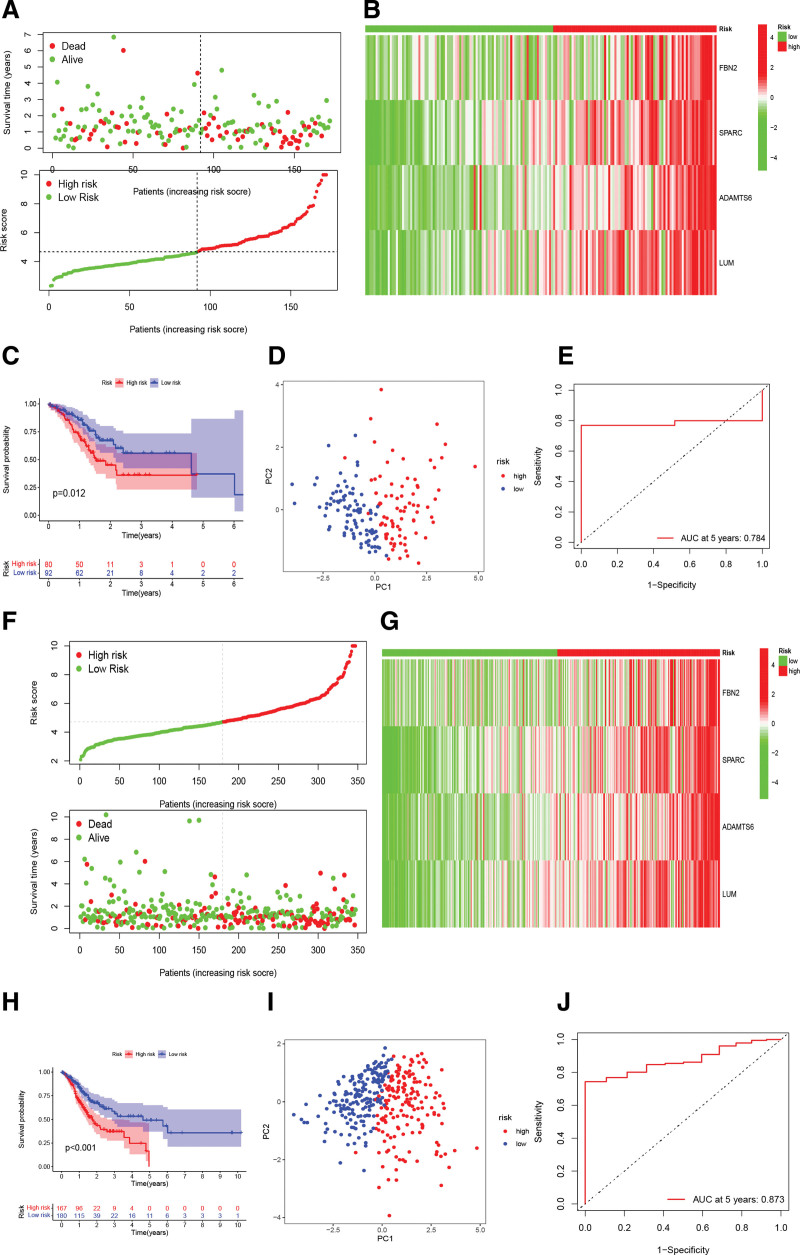
Validation of the accuracy and reliability of the 4-BMRGs signature in the validation set and the overall study cohort. Scatter plots showing the relationship between RS and survival in validation set (A) and overall cohort (F). Heat map of the distribution of 4-BMGRs in validation set (B) and overall cohort (G). Kaplan–Meier survival curves for different risk subgroups in validation set (C) and overall cohort (H). Validation set (D) and overall cohort (I) PCA analysis. ROC curves in validation set (E) and overall cohort (J). BMRGs = basement membrane related genes, ROC = receiver operating characteristic, RS = risk score.

Patients were divided into high- and low-risk subgroups based on the median value of the training set RS as the cutoff point. PCA analysis shows that RS can excellently classify patients into 2 subtypes (training sets: Fig. 4C, validation sets: Fig. 5D). The K-M curves showed worse OS in both the training set (logrank test: *P* = .002, Fig. 4E) and the validation set for the high-risk subgroup (logrank test: *P* = .012, Fig. 5C). ROC curves were plotted for 5 years with areas under the curve (AUC) values of 0.922 and 0.784 for the training and validation cohorts, respectively (Figs. 4D and 5E). Scatter plot showing gradual increase in patient mortality with increasing RS in both the training and validation sets (Figs. 4F and 5A). The heatmap shows high expression of 4-BMRGs in patients with high RS (training sets: Fig. 4G, validation sets: Fig. 5B).

### 3.4. Predictive independence of the BMRGs signature

According to Figure 5F, in the TCGA cohort, the mortality rate of the patients increased with the elevated RS. Additionally, PCA analysis demonstrated that the RS could effectively differentiate patients (Fig. 5I). In the TCGA cohort, 4-BMRGs exhibit significantly higher expression in patients with high RS, as depicted in the heatmap shown in Figure 5G. The AUC values for the TCGA cohort of GC patients at 5 years were 0.873 (Fig. 5J). To verify whether RS has prognostic value independent of clinicopathological indicators (e.g., age, pathological stage, and gender), cox regression analysis and ROC analysis were performed on overall cohort. K–M analysis, univariate and multivariate cox regression analyses showed RS as an independent prognostic factor and the AUC values were superior to clinicopathological indicators (Fig. 5H and Fig 7A, B, E).

### 3.5. Predictive ability to validate risk scores in external cohorts

We tested the reproducibility of the ability to predict prognosis based on the 4-BMRGs signature in the GEO cohort, and A meta cohort consisting of GSE62254, GSE15459 and GSE34942 was served as the external validation set. The results showed that patients in the high-RS group had a significantly shorter survival time than those in the low-RS group (Figure S1C, Supplemental Digital Content, http://links.lww.com/MD/J839). Scatter plot showing gradual increase in patient mortality with increasing RS (Figure S1A, Supplemental Digital Content, http://links.lww.com/MD/J839). The ROC curve for the 5-year survival prediction is depicted in Figure S1B, Supplemental Digital Content, http://links.lww.com/MD/J839.

### 3.6. clinicopathological features

**Figure 6.**
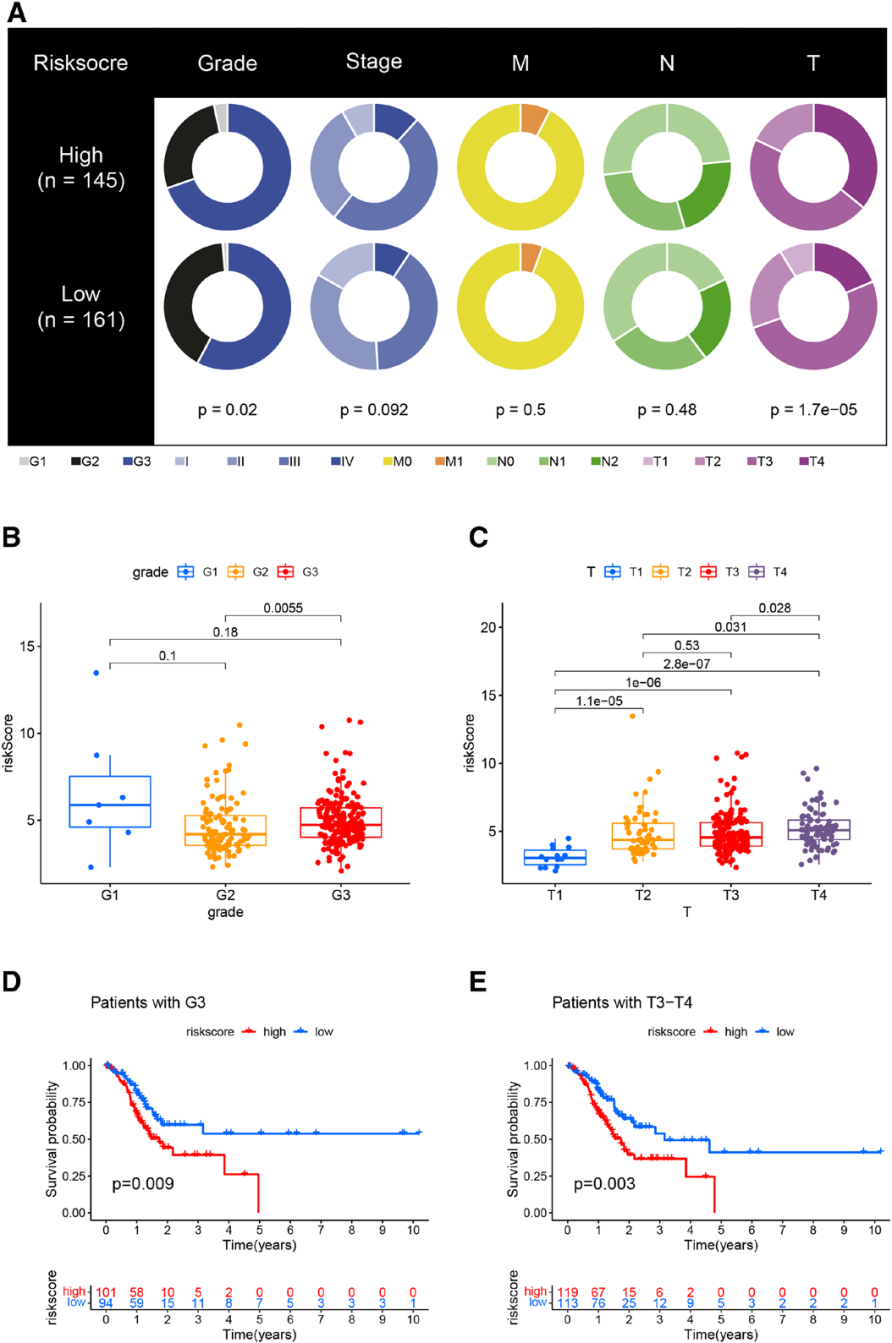
(A) Pie chart demonstrating the association between different RS stratifications and multiple clinicopathological factors. (B) Relationship between different grade and risk scores. (C) Relationship between different T stages and risk scores. (D) Survival analysis between different risk scores for G3 patients. (E) Survival analysis between different risk scores for patients with T3 and T4 stages.

Furthermore, the clinicopathological characteristics of the different RS groups in the TCGA cohort were analyzed. There were significant differences in grade and tumor size between the different RS groups (Fig. 6A). High RS was significantly associated with histological grade 3 and T4 (Fig. 6B and C); and high RS had a worse prognosis in patients with G3 or T3-T4(Fig. 6D and E).

### 3.7. Construction and evaluation of nomogram.

**Figure 7.**
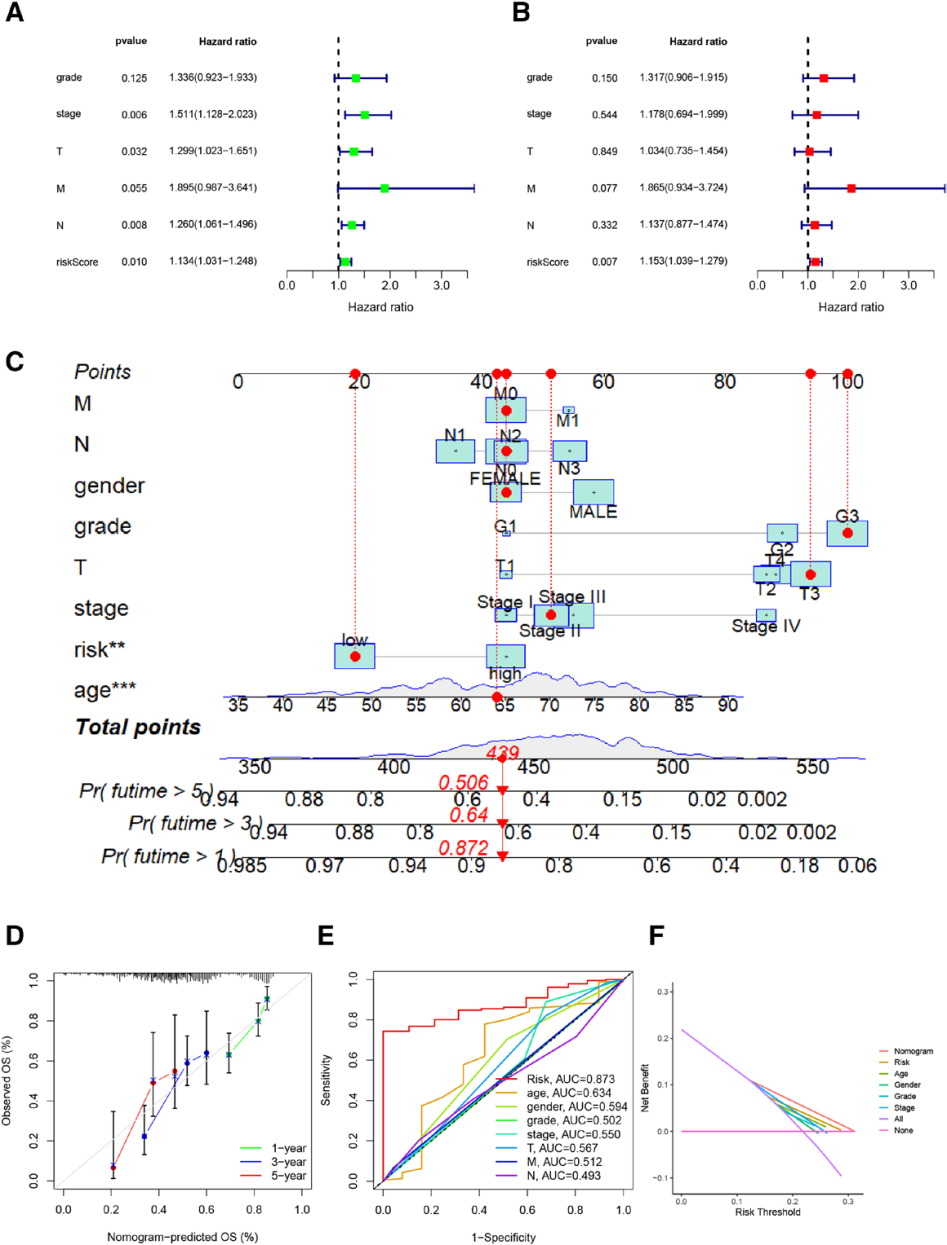
Univariate (A) and multivariate (B) Cox regression in the whole study cohort Nomogram creation and validation. (C) Nomogram. (D) Calibration curves. (E) ROC curves of risk scores and clinicopathological factors. (F) DCA curve. DCA = decision curve analysis, ROC = receiver operating characteristic.

The nomogram was constructed based on RS and clinicopathological factors in the complete cohort to enable the prognostic signature to more accurately predict 1-, 3-, and 5-year OS in GC patients (Fig. 7C). Both the calibration curve and the DCA curve indicate that the nomogram has excellent reliability and yield in prognosis prediction of GC patients (Fig. 7D and F).

### 3.8. Functional enrichment analysis

**Figure 8.**
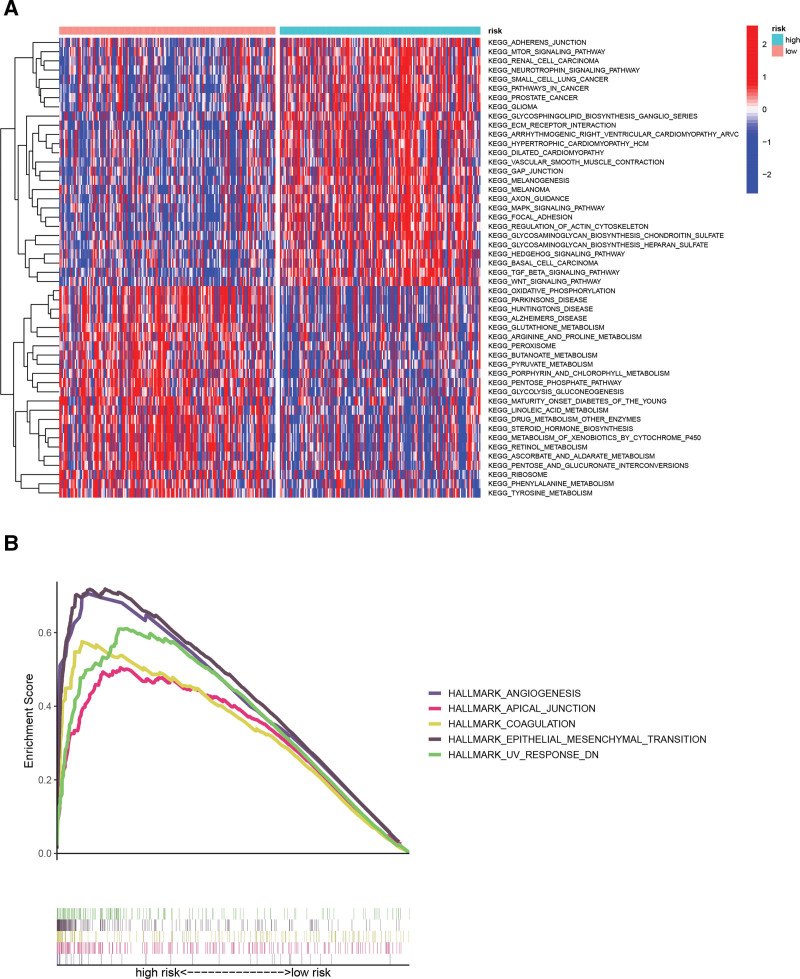
(A) Heatmap illustrating the differential pathways between high- and low-risk subgroups derived from GSVA analysis. (B) GSEA analysis. GSEA = Gene Set Enrichment Analysis, GSVA = gene set variation analysis.

The GSEA analysis was performed to further explore the reasons for the differences in prognosis among the different risk subgroups. The outcomes showed that the high-risk subgroup was notably enriched in ANGIOGENESIS, APICAL JUNCTION, COAGULATION, and EPITHELIAL MESENCHYMAL TRANSITION (EMT) pathways (Fig. 8B). The GSEA enrichment was interestingly concentrated in the high RS group, while the FDR values in the low RS group were all greater than 0.05.

To further exploit potential differences in biological function between risk stratification, GSVA analysis was performed to identify differences in biological pathway activation status across different RS subgroups. The high-risk subgroup presented enriched oncogenic and stroma-related pathways, including TGF BETA SIGNALING PATHWAY, ECM RECEPTOR INTERACTION (ECM), WNT SIGNALING PATHWAY, MAPK SIGNALING PATHWAY, and FOCAL ADHESION pathways (Fig. 8A). Data from these enrichment analyses may provide valuable targets for GC treatment.

### 3.9. Tumor microenvironment, immune cell infiltration analysis

**Figure 9.**
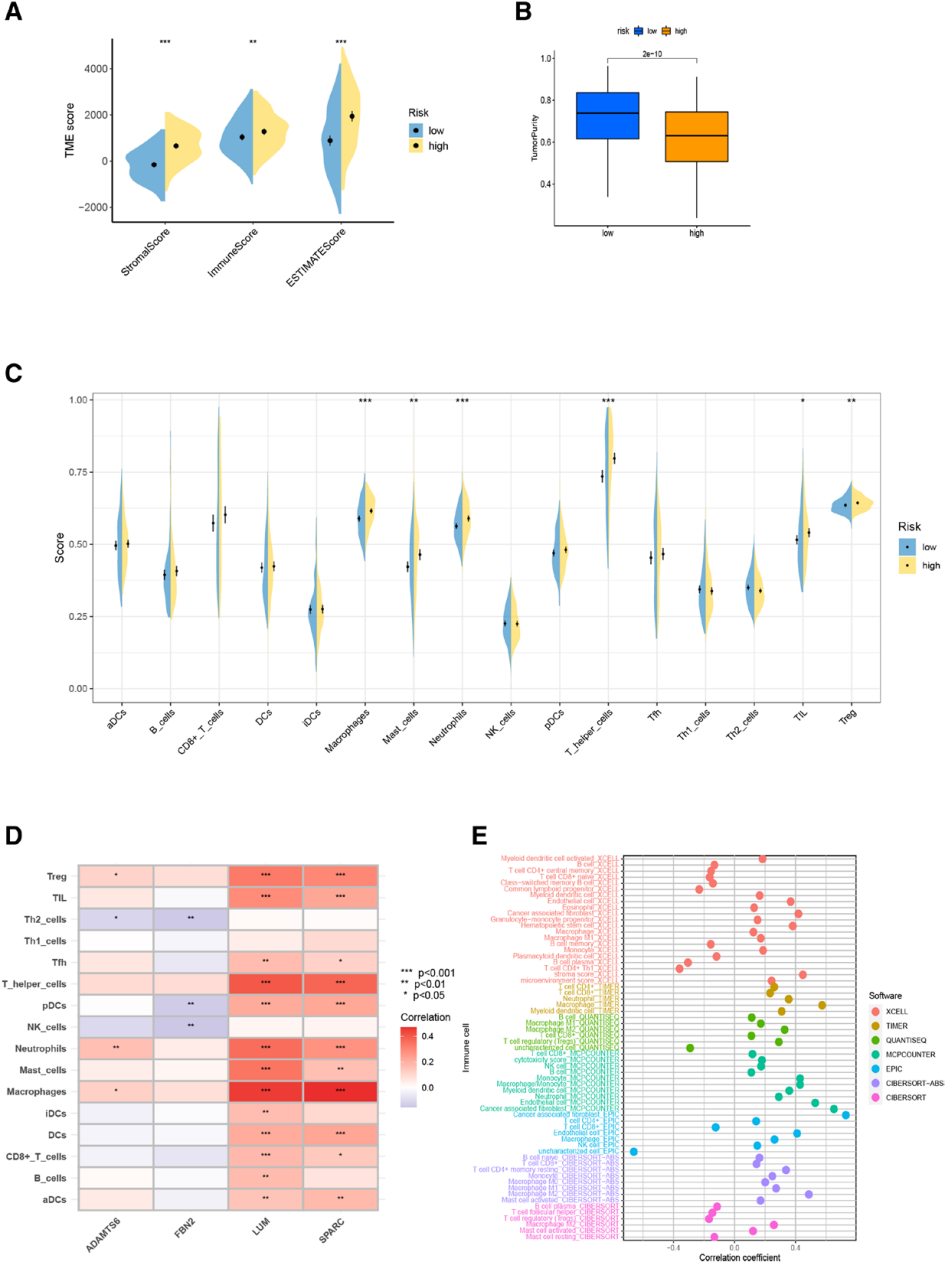
Tumor microenvironment and immune landscape. (A, B) Stromal score, immune score, estimate score, tumor purity. (C) Differences in infiltration of immune cell between risk stratifications. (D) Correlation of 4 BMRGs with immune cell types. (E) Correlation of RS with immune cells. BMRGs = basement membrane related genes, RS = risk score.

Since the high-risk subgroup showed abundant stroma-related signals, we measured the differences in tumor purity, stromal cell distribution, and immune cell infiltration between the different risk subgroups via the “ESTIMATE” algorithm. Figure 9A and B shows that the high-risk subgroup had higher immune and stromal scores, but lower tumor purity.

We estimated the infiltration abundance of immune cells in each GC patient using the ssGSEA. The ssGSEA results showed that Macrophage, Mast cell, Neutrophils, T helper cell, TIL, Monocyte and Tregwere more abundant in the high-risk subgroup (Fig. 9C). Heat map of correlations between immune cells and the 4 BMRGS shows that SPARC and LUM are most strongly correlated with immune cells (Fig. 9D). Risk scores have also been shown to be positively correlated with various immune cells in different algorithmic analyses (Fig. 9E).

### 3.10. Precision medicine and immune infiltration

**Figure 10.**
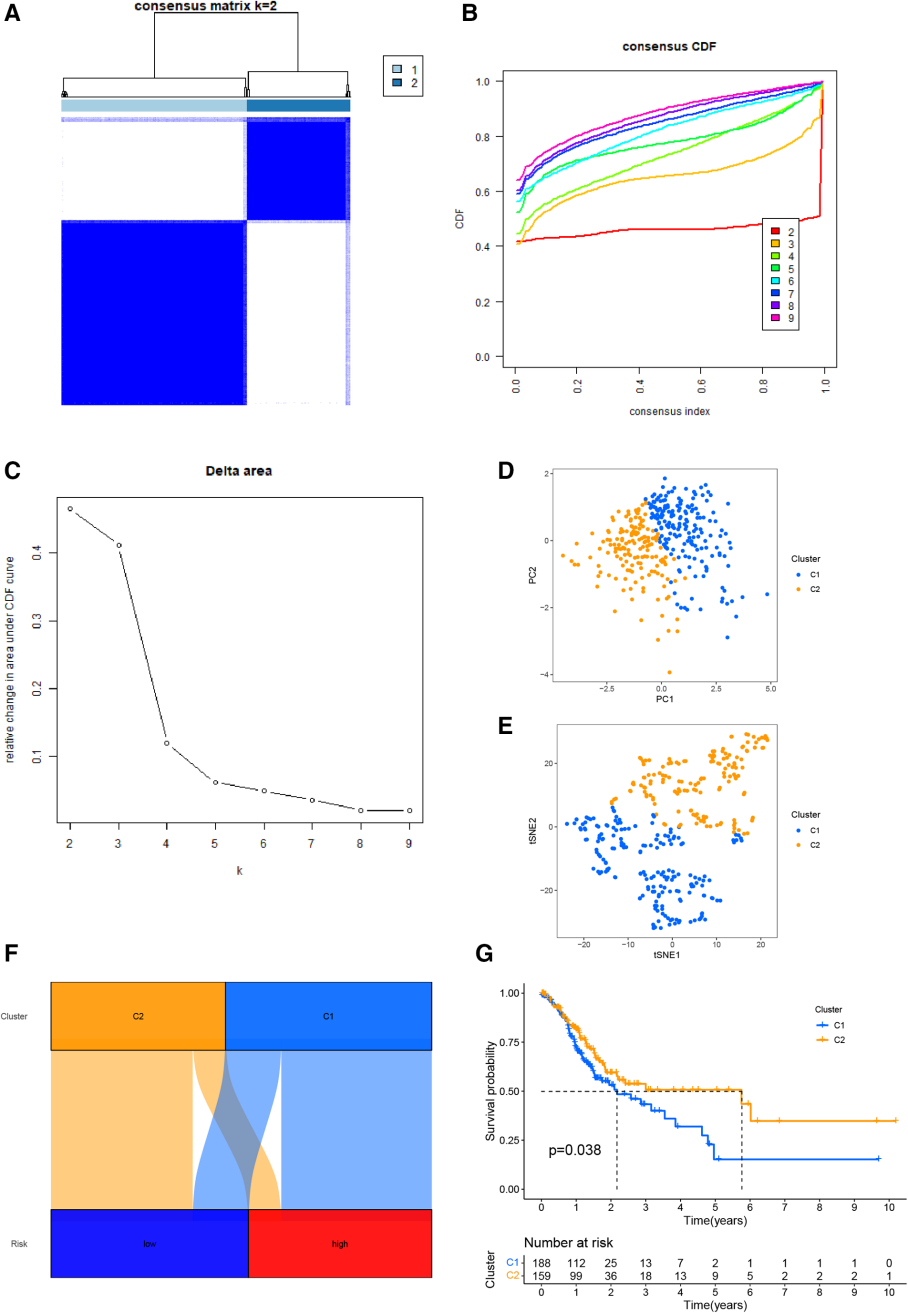
(A–C) Consensus clustering divides patients into 2 molecular subtypes. PCA analysis (D) and tSNE analysis (E). (F) Distribution of patients with different subtypes in high and low risk groups. (G) Survival analysis of different subtypes.

**Figure 11.**
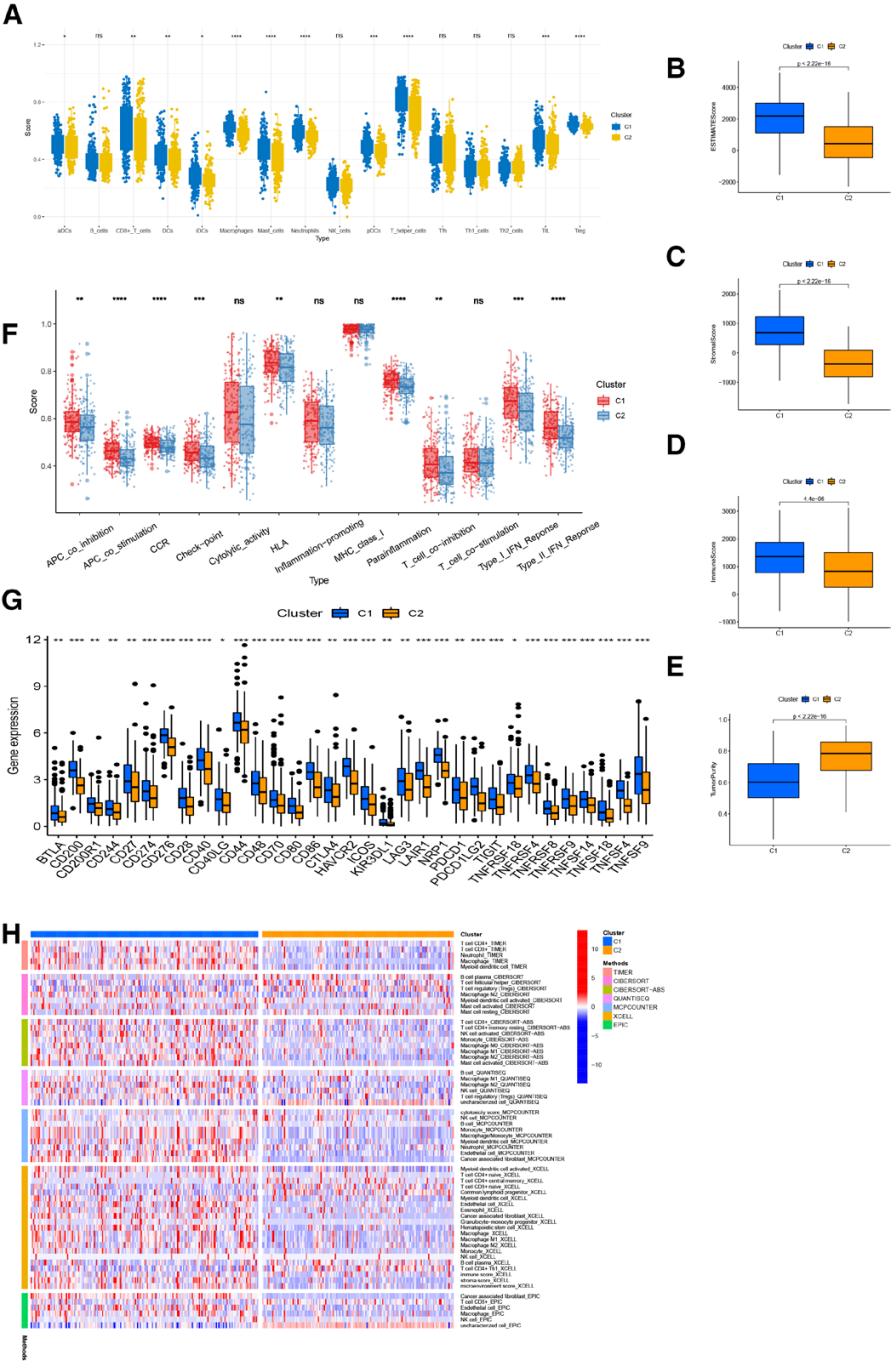
(A) Differences in immune cell infiltration between different molecular subtypes. (B–E) The estimate scores between different molecular subtypes. (F) Differences in immune function between different molecular subtypes. (G) Immune checkpoint differences between molecular subtypes. (H) Heat map of immune cell distribution between molecular subtypes.

To identify the distinct subtypes in STAD, patients were reclassified into 2 clusters based on the 4-BMRGs expression levels in the model using the R package “ConensusClusterPlus” (Fig. 10A–C). The KM curves showed significant differences between the 2 clusters (Fig. 10G). The results of PCA and t-SNE analysis suggest that the 2 clusters have distinct principal components and are significantly different (Fig. 10D and E), the majority of patients in cluster2 being in the low-risk group and the majority of patients in cluster1 being in the high-risk group (Fig. 10F). Previous studies have demonstrated that the immunological microenvironment tends to be different for each subtype of tumor. cluster1 and cluster2 have significant differences in terms of tumor purity, stromal score and immunological score (Fig. 11B–E). The results of ssGSEA and different software analyses show that cluster 1 has a higher abundance of immune cell infiltration (Fig. 11A and H). These results suggest that cluster 1 is an immune “hot tumor,” whereas cluster 2 is a “cold tumor.” There is growing evidence that patients with “hot tumors” are more likely to benefit from immunotherapy. In the analysis of immune checkpoints, CTLA4, HAVCR2, CD274 (PD-L1) and PDCD1 (PD1) were significantly higher in cluster 1 than in cluster 2 (Fig. 11G). Immune function analysis also revealed that cluster 1 scored higher in check point, HLA, MCH I and other functions (Fig. 11F).

### 3.11. Immune checkpoints and immunotherapeutic response

**Figure 12.**
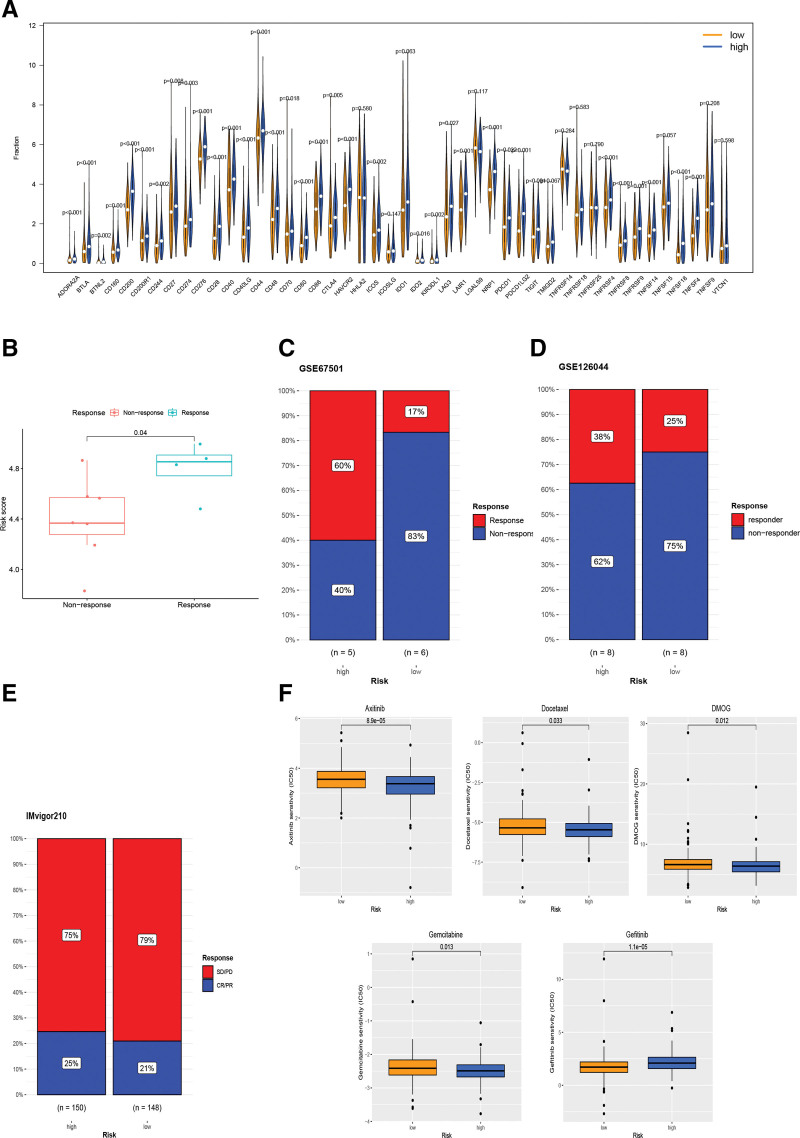
Immunotherapy and chemotherapy. (A) Differences in immune checkpoint between high and low risk groups. (B, C) Immunotherapy response in the GSE67501 data set. (D) Immunotherapy response in the GSE126044 dataset. (E) Immunotherapy response in the imvg210 dataset. (F) Chemotherapeutic drug sensitivity.

We know that immune evasion is essential for tumorigenesis and progression, so we investigated the relationship between RS and immune checkpoint gene expression. As shown in Figure 12A, we found that of the 35 immune checkpoint genes with statistically different expression, 34 were highly expressed in the high-risk subgroup including PD1, PD-L1. Considering that PD-L1 and PD1 differed significantly in different risk scores, we used the aforementioned method to classify the anti-PD1 treatment cohorts GSE67501, GSE126044 and the anti-PD-L1 treatment cohort IMvigor210 into high- and low-risk groups for comparison of immunotherapy efficacy (Fig. 12C–E). Among patients treated with both anti-PD1 and PDL1 we found that the proportion of patients responding was higher in the high-risk group than in the low-risk group and was significantly different in the GSE67501 cohort (Fig. 12B).

### 3.12. Chemotherapy drug efficacy prediction

We evaluated IC50 values for 9 common chemotherapeutic agents per patient and observed significant differences in response to 5 anticancer drugs between 2 risk subgroups (Fig. 12F). The low-risk subgroup of patients had lower IC50 values for Gefitinib. Axitinib, DMOG, Gemcitabine and Docetaxel were more sensitive in the high-risk subgroup. The other 4 chemotherapeutic agents showed no significant sensitivity differences between 2 risk subgroups (Figure S2, Supplemental Digital Content, http://links.lww.com/MD/J840).

### 3.13. RS pan-cancer analysis

**Figure 13.**
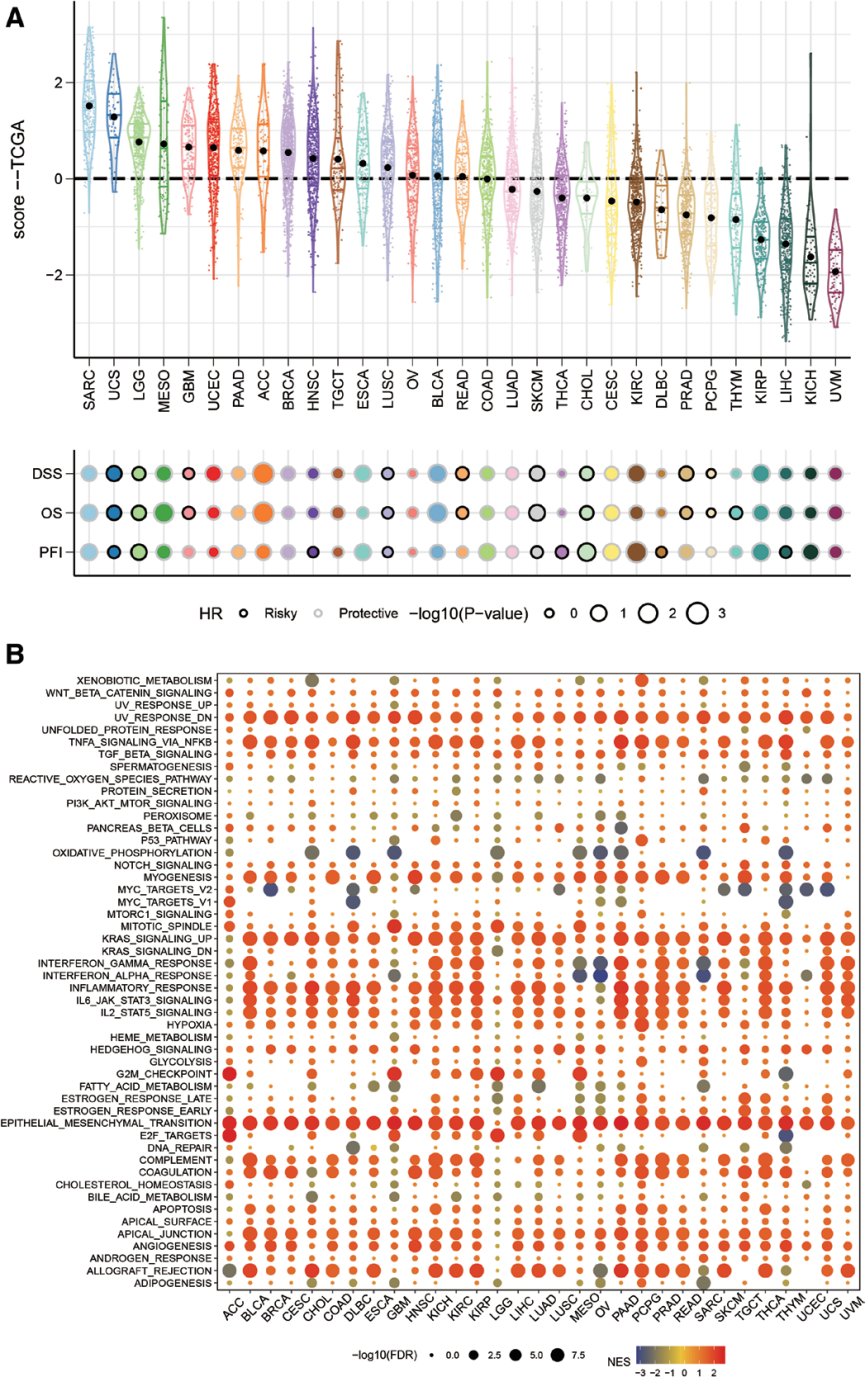
RS score pan-cancer analysis: (A) Risk score ranking and prognosis analysis for 31 cancers. (B) GSEA analysis of differential genes for high and low risk groups for 31 cancers. GSEA = Gene Set Enrichment Analysis, RS = risk score.

Analysis of 31 cancers according to the RS formula revealed that the RS were relatively high in SARC, UCS, and LGG. and was a prognostic factor for most cancers (Fig. 13A). The top 30% and bottom 30% of patients with risk scores for each cancer were analyzed for differences, and GSEA was performed with the difference genes. The results showed that RS was closely related to KRAS signaling up, EMT and other pathways related to cancer development and metastasis, and the immune-related pathways IL6 JAK STAT3 signaling and IL2 STAT5 signaling were significantly different in patients with high and low RS (Fig. 13B).

## 4. Discussion

As one of the most aggressive malignant tumors, gastric cancer often metastasizes at an early stage.^[19]^ It has been confirmed that tumor metastasis is often accompanied by destruction of the basement membrane.^[20]^ Therefore, the basement membrane is critical to the prognosis of gastric cancer. To identify the most representative survival-related BMRGs, univariate Cox analyses were performed to accurately filter BMRGs that were valuable for GC prognosis. The least absolute shrinkage and selection operator regression analysis was used to identify hub BMRGs, and a prognostic signature with 4 BMRGs was finally constructed (FBN2, ADAMS6, SPARC, LUM). KM curves, ROC curves, nomogram, calibration curves and DCA validated the excellent clinical applicability of the 4-BMRGs signature, which can effectively predict the prognosis of gastric cancer patients. Moreover, the 4-BMRGs signature can also predict the immune infiltration status of gastric cancer and its responsiveness to immunotherapy. Furthermore, the 4-BMRGs signature can accurately predict the sensitivity of gastric cancer patients to different chemotherapy and targeted drugs.

Although there have been some models^[21–23]^ for predicting the prognosis of gastric cancer patients, the high heterogeneity of gastric cancer makes the existing prognostic models still insufficient for clinical needs. Furthermore, the basement membrane plays a crucial role in the prognosis of gastric cancer, but in-depth research is currently lacking. The strength of this study lies in comprehensive bioinformatics analysis, exploring the impact of basement membrane genes on the prognosis of gastric cancer patients and revealing their relationship with the immune microenvironment and drug treatment response. Wang et al identified a prognostic model containing 6 BMRGs to predict the prognosis of gastric adenocarcinoma patients, with an AUC value of 0.7 for 5-year overall survival (OS).^[24]^ In contrast, our model achieved a significantly higher AUC value of 0.873 for predicting 5-year OS in gastric adenocarcinoma patients, outperforming Wang et al’s model. Moreover, our model includes 4 key genes that are entirely different from the 6 key genes in their model.

Tumour formation, overgrowth and metastasis comprise a complex series of cellular and molecular events that result from a combination of aberrant signaling pathways, mutations and epigenetic alterations. Enrichment analysis of 32 prognosis-related basement membrane genes showed that BMRGs is involved in many important structures, such as extracellular matrix organization, collagen-containing extracellular matrix, and extracellular matrix structural constituent. It indicates that the above genes are inextricably linked to the extracellular matrix and are important influences on cancer metastasis.^[25,26]^ In the KEGG analysis BMRGs were associated with several critical neoplasm-related pathways, such as the PI3K-Akt, ECM-receptor interaction pathway. PI3K-Akt signaling pathway has been demonstrated to be overactive in various cancers.^[27]^ Recent studies have revealed that basement membrane-associated components promote cancer metastasis through activation of PI3K.^[28]^ Among these 4-BMRGS, the fibronectin 2 molecule is one of the structural cores of microprotofibrils. It was found that a dysregulated ratio of FBN2 to FBN1 may stimulate tumor angiogenesis by affecting the activation of TGF-β.^[29]^ Lumican is a ubiquitous ECM component whose expression is significantly upregulated in various tumors, particularly in the stomach,^[30]^ colon^[31]^ and lung.^[32]^ Lumican was found to be overexpressed by cancer-associated fibroblasts (CAF) in the gastric cancer tumor microenvironment.^[33]^ Lumican highly expressed in CAFs promotes cancer growth by activating the integrin β1-mediated FAK signaling pathway and is positively associated with poor clinical prognosis.^[33]^ SPARC, an extracellular matrix glycoprotein, plays a role in tissue remodeling and cell-matrix crosstalk. Zhao et al found that SPARC was highly expressed in 436 gastric cancer patients and was associated with poor prognosis in gastric cancer.^[34]^ ADAMTS (a disintegrin and metalloprotease with thrombospondin motifs) is a secreted multidomain matrix-associated zinc metalloendopeptidase that plays an important role in controlling the structure and function of the ECM.^[35]^ ADAMTS6, a member of the ADAMTS family, has recently been reported to be overexpressed in esophageal and gastric cancers and is associated with poor prognosis.^[36,37]^ Increased expression of ADAMTS6 promotes cancer metastasis.^[38]^ To further dig into the underlying mechanisms of survival differences among different risk subgroups, we performed GSVA as well as GSEA analysis on them. The results indicated that the high-risk subgroup was significantly enriched in cancer-related pathways such as EMT, angiogenesis, apical junction, and coagulation. Previous studies have shown that EMT promotes the migration and invasion of gastric cancer cells. For example, tumor-associated neutrophils can induce EMT in gastric cancer cells through IL-17a, thereby promoting their migration and invasion.^[39]^ The downregulation of miRNA-214 in tumor-associated fibroblasts can also induce EMT and promote the migration and invasion of gastric cancer cells.^[40]^ Angiogenesis is one of the key factors supporting tumor development, where various angiogenic factors are often overexpressed to facilitate the formation of new blood vessel networks and increase the risk of tumor cell metastasis, resulting in poor prognosis.^[41,42]^ Takahashi et al^[43]^ found that enhanced apical junction pathway in gastric cancer is associated with increased potential for metastasis and poorer clinical outcomes.Tumor development, growth and metastasis are often accompanied by a hypercoagulable state, pathological angiogenesis and more. The ability of BM to bind and store growth factors promotes rapid pathological hematologic reconstitution, and ADAMTS involved in basement membrane proteoglycan and glycoprotein modifications are also relevant to coagulation.^[44–46]^

Immune cell infiltration in tumor microenvironment has an important impact on cancer progression.^[47]^ basement membrane has key roles in regulating the activity and function of immune cells in the tumor microenvironment.^[48]^ Further analysis of immune infiltration revealed that Macrophage, Mast cells, Neutrophils, T helper cells, TIL, Monocyte and Treg differ significantly in high and low risk. It has been demonstrated that M2 macrophages might promote tumor cell proliferation through ornithine and polyamine synthesis pathways.^[49]^ In addition, M2 macrophage-mediated immunosuppression is also an important cause of tumor progression.^[50]^ Monocytes facilitate carcinoma development by exerting biological processes such as pro-angiogenesis and lymphadenopathy, and metastasis.^[51]^ These findings suggest that the interaction of immune cells with tumor cells and the extracellular matrix in the tumor microenvironment may contribute to the poor prognosis in the high-risk subgroup. Neoplastic cells usually evade immune cells by exploiting the properties of immune checkpoints.^[52]^ Since immune checkpoints have a significant role in tumor immune escape, we examined the differences in common immune genes between high and low risk subgroups. We found that most immune checkpoint genes, including PD1 and PD-L1, are highly expressed in the high-risk subgroup, and their overexpression inhibits the normal function of immune cells, thereby promoting tumor progression.^[53]^ This suggests that immune suppression mediated by inhibitory immune checkpoints contributes to unfavorable outcomes in the high-risk subgroup. Immune checkpoint inhibitors (ICI) achieve anti-tumor effects by unlocking the suppressive effect of tumor cells on the immune system and restoring the recognition and killing of tumor cells by T cells.^[54]^ Considering the effectiveness of ICI in cancer treatment, we assessed the sensitivity of GC patients to immunotherapy using anti-PD1 and anti-PDL1 immunotherapy cohorts. The immunotherapy response rate was higher in the high-risk subgroup than in the low-risk subgroup, suggesting the efficacy of ICI for them, suggesting that ICI may have limited efficacy for them. These results could be helpful for personalized treatment of GC patients. Although surgery still has a pivotal role in the treatment of gastric malignancies, however, among the systemic treatments for GC, chemotherapy and immunotherapy can have an unparalleled benefit in patient survival.^[55]^ Our study compared the sensitivity of 9 common chemotherapeutic agents in different risk subgroups. Axitinib, DMOG, Gemcitabine and Docetaxel were found to have better efficacy in the high-risk subgroup, while Gefitinib was more sensitive in low-risk patients.

We determined the stability of the 4-BMRGs signature by internal and external validation in the TCGA database, but our study has several limitations. First, our study population consists mainly of GC samples from publicly available TCGA and GEO databases, and we still need to confirm the accuracy of the BMRGs signature in real-world gastric cancer cohorts. Second, the detailed mechanisms of some BMRGs in GC are unclear and require elucidation by in vivo and in vitro experiments. Finally, as a retrospective study, selection bias and introduction bias cannot be avoided and the sample size is relatively small. Therefore, a higher level of evidence as well as larger data are needed to confirm the results of this study.

In brief, we constructed a signature consisting of 4-BMRGs, which exhibited accuracy and reliability in predicting GC patients’ prognosis. Besides, we performed tumor microenvironment analysis, immune landscape analysis, drug prediction and immunotherapy response analysis among different risk subgroups. These findings could contribute to personalized treatment strategies for GC.

## Author contributions

**Conceptualization:** Zhiyang Liu.

**Methodology:** Zhiyang Liu..

**Supervision:** Lin Xin.

**Writing – original draft:** Zhiyang Liu.

**Writing – review & editing:** Zhiyang Liu.

## Supplementary Material






