# Supplementary figures and images for: Identification of a basement membrane-related genes signature to predict prognosis, immune landscape and guide therapy in gastric cancer
Source: Medicine (Baltimore). 2023 Sep 29;102(39):e35027. doi: 10.1097/MD.0000000000035027 (PMC10545384; doi:10.1097/MD.0000000000035027)

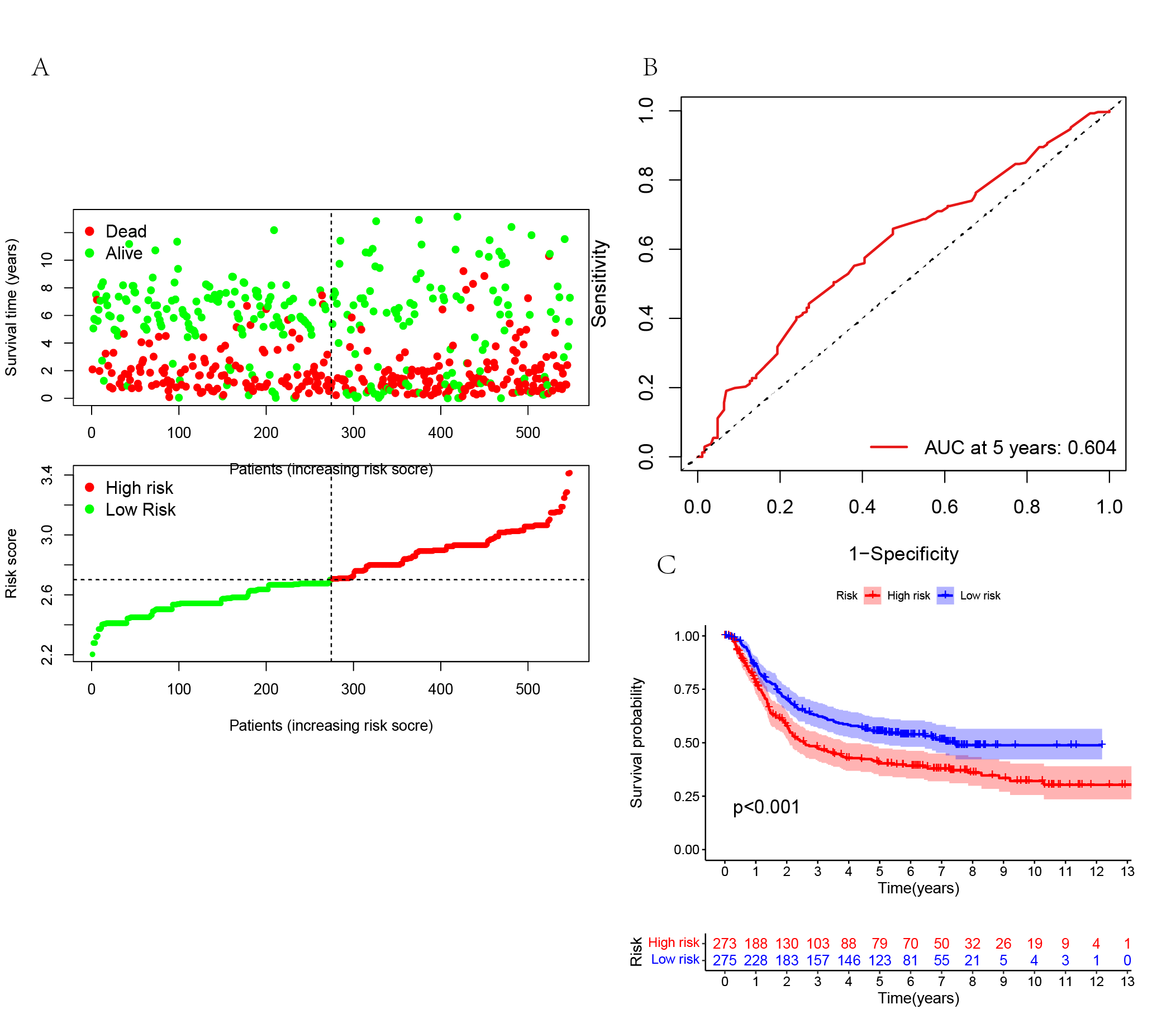

Supplement: Supplementary file 2 [file medi-102-e35027-s002.tif]

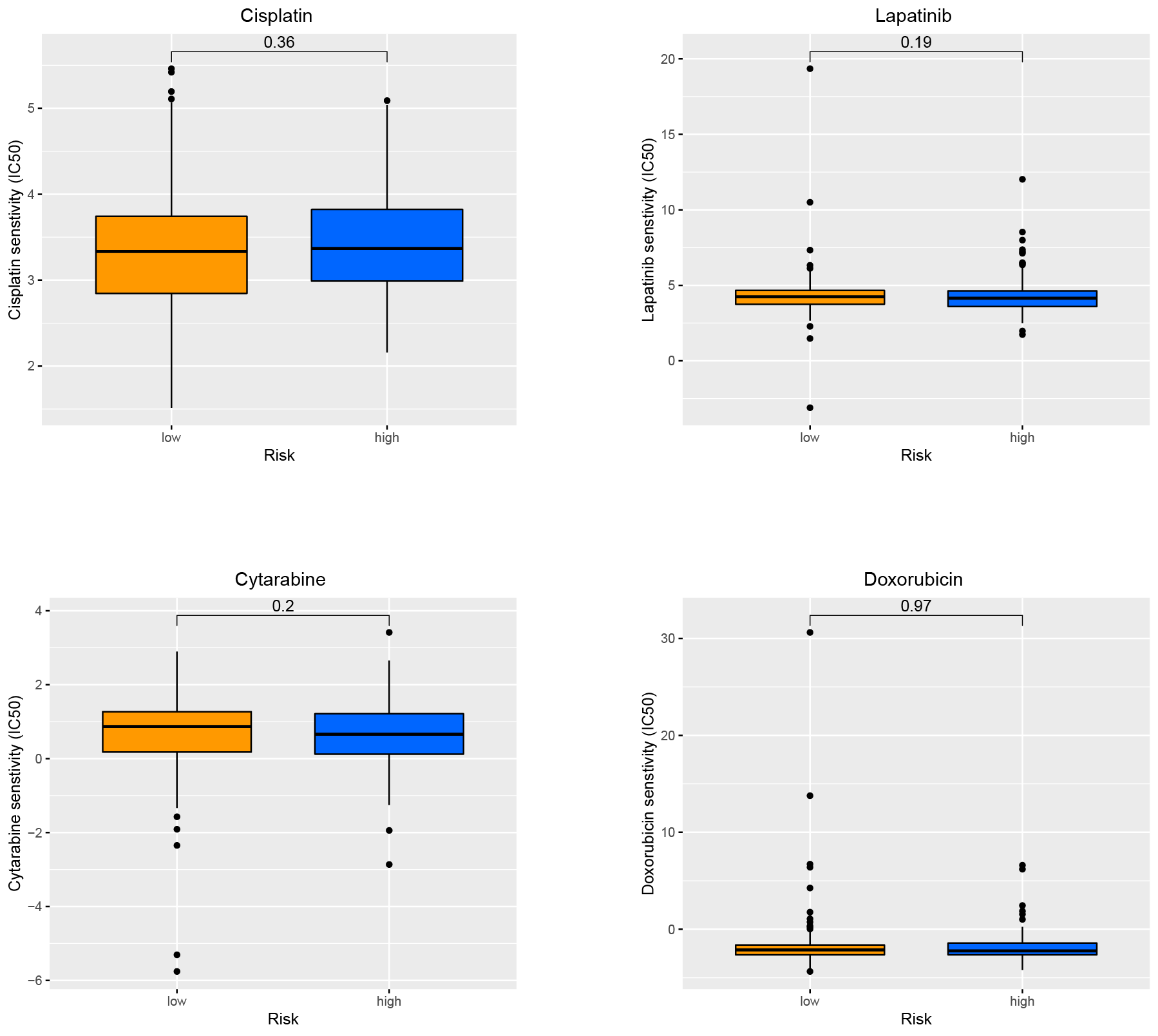

Supplement: Supplementary file 3 [file medi-102-e35027-s003.tif]
